# Investigating the impact of external load on muscle synergies during bipedal squats

**DOI:** 10.1007/s00421-024-05432-3

**Published:** 2024-02-21

**Authors:** Rouven Kenville, Martina Clauß, Tom Maudrich

**Affiliations:** 1 Department of Movement Neuroscience, Faculty of Sports Science, Leipzig University, 04109 Leipzig, Germany; 2 Department of Neurology, Max Planck Institute for Human Cognitive and Brain Sciences, 04103 Leipzig, Germany; 3 Faculty of Sports Science, Leipzig University, 04109 Leipzig, Germany

**Keywords:** Bipedal squat, Functional movements, Muscle synergies, Surface electromyography

## Abstract

**Purpose:**

A broad functional movement repertoire is crucial for engaging in physical activity and reducing the risk of injury, both of which are central aspects of lifelong health. As a fundamental exercise in both recreational and rehabilitative training regimes, the bipedal squat (SQ_Bp_) incorporates many everyday movement patterns. Crucially, SQ_Bp_ can only be considered functional if the practitioner can meet the coordinative demands. Many factors affect coordinative aspects of an exercise, most notably external load. Since compound movements are assumed to be organized in a synergistic manner, we employed muscle synergy analysis to examine differences in muscle synergy properties between various external load levels during SQ_Bp_.

**Methods:**

Ten healthy male recreational athletes were enrolled in the present study. Each participant performed three sets of ten SQ_Bp_ on a smith machine at three submaximal load levels (50%, 62.5%, and 75% of 3 repetition maximum) across three non-consecutive days. Muscle activity was recorded from 12 prime movers of SQ_Bp_ by way of electromyography (EMG). Muscle synergies were analyzed in terms of temporal activation patterns, i.e., waveform, as well as the relative input of each muscle into individual synergies, i.e., weight contribution.

**Results:**

Waveforms of muscle synergies did not differ between loads. Weight contributions showed significant differences between load levels, albeit only for the gastrocnemius muscle in a single synergy.

**Conclusion:**

Taken together, our results imply mostly stable spatiotemporal composition of muscle activity during SQ_Bp_, underlining the importance of technical competence during compound movement performance in athletic and rehabilitative settings.

## Introduction 

Compound movements such as the bipedal squat (SQ_Bp_) play an important role in the management of everyday motor activities. SQ_Bp_ comprises many movement patterns of everyday life (Nelson et al. 2002), while representing an integral part of lower extremity muscle strength training programs in both amateur and competitive athletes (Slater and Hart 2017). Moreover, SQ_Bp_ is employed in the rehabilitative field, e.g., to rebuild muscle mass and strength after injury (van Rossom et al. 2018), as well as for fall prevention in the elderly (Rosendahl et al. 2008). When using SQ_Bp_ to improve performance, learning proper technique precedes the application of external load, due to the high coordinative demands of this exercise (Clark et al. 2012). Once technique is established, the emphasis can be placed on a progressive increase in load to build muscle strength and mass. Consequently, in both athletic and rehabilitative fields, the quality of performance determines its suitability, meaning SQ_Bp_ can only be classified as useful or functional if the person exercising possesses the necessary coordinative abilities.

Coordinative demands within SQ_Bp_ relate to multiple parameters such as dynamic adjustments of forces and torques (Dionisio et al. 2008), maintaining an optimal body alignment and stability (Garland et al. 2009), as well as the control of concomitant movements (movements not performed by prime movers) (Cordo and Gurfinkel 2004). Furthermore, it is important to consider that movements progress through different movement periods, i.e., periods of concentric, isometric, and eccentric muscle actions, which means that movement parameters change dynamically throughout each repetition cycle (Duchateau and Baudry 2014; Duchateau and Enoka 2008). For these reasons, processing within the central nervous system (CNS) must meet all coordinative challenges to ensure optimal SQ_Bp_ performance. Given the associated computational magnitude, reducing its complexity appears to be a useful goal of CNS-moderated motor control (Bernstein 1967). Indeed, it has been demonstrated in animals that the CNS can evade complexity of motor control mechanisms by generating motor commands through a linear combination of muscle synergies (d'Avella et al. 2003). In short, muscle synergies reflect systematic patterns of muscle activity encoded within certain neural sites (Rana et al. 2015). A single muscle synergy can generate a specific motor action, and it is assumed that combining muscle synergies in a flexible manner elicits a broad range of motor outputs (d'Avella et al. 2003). In humans, muscle synergies can be studied non-invasively through the analysis of multi-muscle electromyography (EMG) data. Using techniques such as non-negative matrix factorization (NNMF), it is possible to deconstruct largescale EMG data into less complex components that may ultimately be related to specific actions within a movement (Turpin et al. 2021). Previous research demonstrated that the variance of multi-muscle EMG activity during compound motor actions such as running (Cappellini et al. 2006) and cycling (Hug et al. 2011) can be explained by a few muscle synergies. Coordinative demands can also be assessed through muscle synergy analysis by comparing the number and properties of individual synergies between conditions during movement execution (Turpin et al. 2011). For instance, Smale et al. (2016) observed differences in the number of muscle synergies between two coordinatively varying types of squat movements (uni- and bipedal execution), highlighting the importance of considering the impact of basic control variables (e.g., exercise variations, load, volume, number of repetitions and sets, tempo, and break periods) on coordinative demands within SQ_Bp_ to match performance levels with exercise demands.

A commonly employed control variable to progressively advance the induction of resistance training objectives such as hypertrophy and muscle strength in both rehabilitative and competitive settings is external load (Lopez et al. 2021). Previous research in both simple (Coscia et al. 2014; Roh et al. 2019) and compound movements (Hug et al. 2011; Turpin et al. 2011) suggests that muscle synergies are unaffected by external load. Critically, a systematic investigation of such effects during compound movement performance under standard training conditions, used in both recreational and rehabilitative settings, remains elusive. For this reason, our goal was to investigate the influence of external load on muscle synergy properties within SQ_Bp_ to evaluate coordinative demands as a function of external load. Based on previous studies, we hypothesized stable muscle synergy properties across varying submaximal load levels. With this study, we aimed to describe muscle synergies during SQ_Bp_ performance and to provide insight into the coordinative demands of SQ_Bp_ at different load levels. We intend our findings to support the design of individualized training recommendations pertaining to the relationship between technique and load in both rehabilitation and competitive sport contexts.

## Materials and methods 

This study partly represents a reanalysis of previously published sEMG data (Maudrich et al. 2022).

### Participants

Ten healthy male recreational athletes (aged 24.5 ± 3.3 years (mean ± SD), body mass 82.2 ± 7.1 kg, height 183.5 ± 5.1 cm, weight training experience 3.0 ± 2.8 years) were included in this study. Two participants from our previous analysis had to be excluded due to missing or incomplete EMG data for lower leg muscles*,* which were additionally analyzed in the present analyses. Participants were informed about the procedures as well as possible risks and benefits and confirmed their participation by signing an informed consent form in accordance with the Declaration of Helsinki. The local ethics committee of Leipzig University approved this study (ref.-nr. 271/21-ek).

### Procedures

**Fig. 1:**
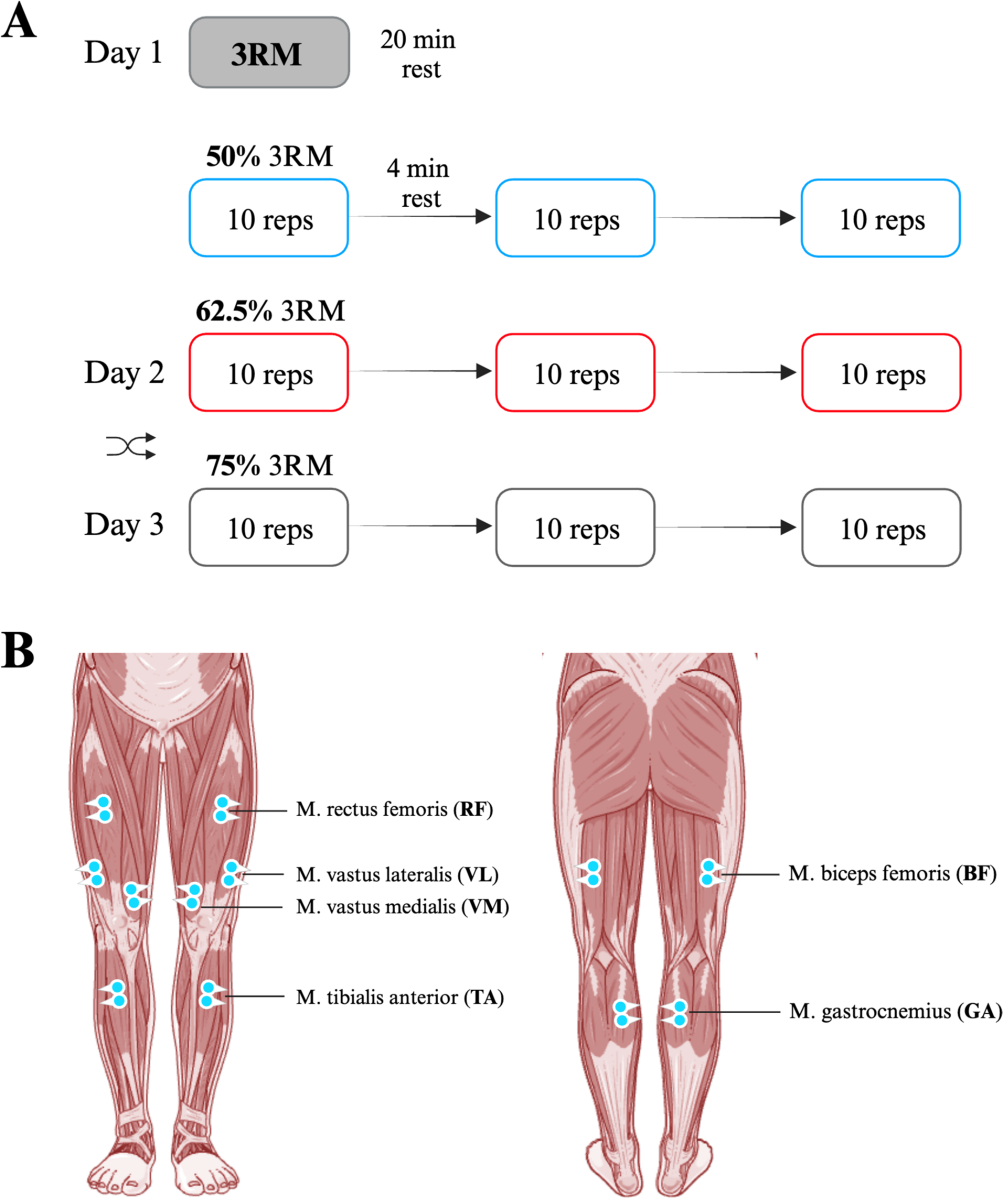
Experimental protocol. **A** Each participant performed standardized SQ_BP_ on a Smith machine at three loads: 50%, 62.5%, and 75% of the individual three-repetition maximum (3-RM). All load conditions were performed on different days, separated by 1 week. Each testing day consisted of three sets of ten repetitions, with a 4-min rest period in between sets (**B**) EMG activity of M. gastrocnemius caput mediale (GA), M. biceps femoris (BF), M. rectus femoris (RF), M. vastus medialis (VM), M. vastus lateralis (VL), and M. tibialis anterior (TA) was recorded bilaterally

Each participant performed standardized SQ_BP_ on a Smith machine (Technogym Germany GmbH, Germany) at three loads: 50%, 62.5%, and 75% of the individual three-repetition-maximum (3-RM). The 3-RM of a person is the maximum weight they can lift three times (Haff and Triplett 2015). All load conditions were performed on different days, separated by one week (see Fig. 1A). Participants were also told not to perform lower extremity strength training prior to the testing days. Each testing day consisted of 3 sets of 10 repetitions, with a 4-min rest period in between sets (Haff and Triplett 2015). SQ_BP_ execution was standardized according to the following criteria. We first determined the individual knee flexion angles (mean: 70 ± 7.5°) equivalent to the position in which the upper thigh was parallel to the floor. To this end, all participants slowly moved down in the smith machine until they reached the required position. All participants were instructed to (a) keep both feet in contact with the ground during SQ_BP_ and (b) maintain a slight external rotation of both feet at the same time. Furthermore, we ensured that knee and ankle were in line. We then used a digital protractor to identify individual knee angles in this position. A custom-built Laser Sharp^®^ IR sensor (Sharp Business Systems Deutschland GmbH, Germany) attached to the smith machine tracked the progression of the vertical bar position over time, while angular trajectories were calculated from the resulting data. Angular trajectories were subsequently synced to an auditory feedback device, where a tone was generated as soon as the predetermined knee angle was reached during each repetition. In this way, we maintained a standardized range of motion within and between participants. To keep a constant time, one SQ_BP_ movement consisted of three successive movement periods, descent, ascent, and hold, each lasting 2 s. The onset of each movement period was visually signaled via a monitor positioned in front of the participants. Finally, stance was set at shoulder width with both feet turned outward by 10°. Lastly, participants were instructed to keep their feet grounded throughout SQ_BP_.

The initial testing session was used to establish individual 3-RM values. Following 3-RM determination (mean: 97.5 ± 26.4 kg), participants rested for 20 min to attenuate possible fatigue effects. Thereafter, the SQ_BP_ task was performed with the first load (50% of the 3-RM load). Each participant then completed a further warm-up protocol comprising 5 min on a cycle ergometer (120 W; 75 rpm), followed by a set of ten repetitions at no external load, five repetitions at 50% of the eventual test load (i.e., 50% of 3-RM) and finally three repetitions at 75% of the eventual test load. Lastly, three sets of ten repetitions with 50% of the 3-RM load were performed in the standardized manner described above. The two remaining loads were performed on separate days, according to the same protocol. We randomized the order of subsequent test loads (62.5% 3-RM, 75% 3-RM). Due to the novelty of SQ_BP_ on the Smith machine and the high mechanical as well as metabolic stimulus on the entire body following the exercise protocol, we decided to always begin with the lightest of the three load levels (50% of 3-RM) to ensure a low-risk exercise entry in terms of injury prevention and to minimize the possible effect of fatigue following the 3-RM testing, which was performed first on the initial testing day.

### Data recording

EMG activity of M. gastrocnemius caput mediale (GA), M. biceps femoris (BF), M. rectus femoris (RF), M. vastus medialis (VM), M. vastus lateralis (VL), and M. tibialis anterior (TA) was recorded bilaterally (see Fig. 1B) using a wireless desktop transmission system (NORAXON Inc., Scottsdale, AZ). Optimal signal quality was ensured by shaving, abrading using a fine nail file and cleaning each participant’s skin with alcohol. Self-adhesive electrodes (interelectrode distance of 20 mm) were then placed on standardized electrode positions according to SENIAM recommendations (Hermens et al. 2000). Anatomical landmarks ensured constant electrode positions between sessions. All EMG electrodes were placed in alignment to muscle fiber orientations. Data were recorded at a sampling frequency of 1500 Hz, while the input impedance of the amplifier was set at > 100 MΩ, bandpass filtering was applied in the frequency range of 10–500 Hz, and common-mode rejection (CMRR) was set at > 100 dB. Maximum voluntary contraction (MVC) values were recorded bilaterally for each muscle at the beginning of every single testing session. To determine the MVC of bilateral GA, BF, RF, VM, VL, and TA, two maximal isometric contractions (5 s) were performed for each muscle according to contemporary guidelines using external resistance provided manually by one researcher (Konrad 2005) resulting in a total of 12 MVC values. The MVC value used for amplitude normalization of all trials in a testing session was determined by taking the maximum RMS value of both MVC contractions of each participant for each muscle, separately. A rest period of 30 s separated each MVC trial.

### Preprocessing

First, data were normalized to individual MVC values and separated into three sets of ten repetitions (consecutive descent and ascent periods). Initiation and termination of the movement periods were determined on the basis of the angular trajectories derived from the path of the barbell. Data were subsequently collapsed across repetitions per set yielding EMG time series in a MUSCLE x SET format for each participant per load condition.

### Muscle synergy analysis

Preprocessed EMG data were high-pass filtered at 30 Hz (2^nd^-order Butterworth filter) and rectified using the modulus of the analytic signal. To obtain signal envelopes, we low-pass filtered all data using a second-order Butterworth filter with a cutoff frequency of 10 Hz. Signal envelopes were normalized to their maximum activity during each set and load, respectively, and further normalized to the standard deviation to obtain unit variance (Oshima et al. 2022). Finally, envelopes were time-normalized so that every repetition of SQ_BP_ had the same number of samples and visually inspected by a single trained researcher to remove erroneous trials. Subsequently, 5 ± 2.5 trials (mean ± standard deviation) were removed from further analyses across conditions. Muscle synergies were estimated by way of non-negative matrix factorization (NNMF). Here, we employed NNMF using a multiplicative update algorithm (Lee and Seung 1999). A set of one to ten synergies was extracted iteratively. NNMF was limited to 50 replicates, each with 1000 iterations and a termination tolerance of 10^6^ and 10^4^ for the change in temporal activation patterns, i.e., waveforms, the magnitude of muscle weights, and residual size, respectively (Zandvoort et al. 2019). To determine the number of muscle synergies required, we estimated the reconstruction accuracy (RA) of the EMG envelopes. RA was determined by calculating the ratio of the Frobenius norm of the error and the Frobenius norm of the EMG envelopes. Here, the error was specified as the difference between the EMG envelopes and the product of muscle weights and waveforms (Kerkman et al. 2020). A sufficient number of synergies was reached, when RA exceeded a threshold of 90% (Oshima et al. 2022; Zandvoort et al. 2019) with each additional synergy not increasing RA by more than 3% (Boccia et al. 2018). To enable comparison of weights and waveforms between conditions, weights were normalized to the norm of each weight while each waveform was scaled by the same value (Oshima et al. 2022). This step allows possible differences in waveforms to be attributed to factors other than EMG amplitude variations between loads. We further calculated the relative contribution of each synergy to the extracted waveform and weights based on RA. Again, the ratio between the Frobenius norm of the error and the Frobenius norm of the rectified EMG signal were obtained, but here the error was defined as the difference between the EMG envelopes and the product of muscle weights and temporal activation patterns per synergy (Zandvoort et al. 2019). All muscle synergies were subsequently sorted based on the relative timing of the most prominent peak within each temporal activation pattern (Kerkman et al. 2020).

### Statistical analysis

To simplify the statistical model, waveforms and weights were collapsed across all three sets per synergy. Differences in waveforms were analyzed using statistical parametric mapping (Pataky et al. 2015) including paired *t* tests (www.spm1d.org). All *p* values were adjusted for multiple comparisons.

To compare differences in weight contribution to each synergy, we computed two-way repeated-measures analysis of variance (ANOVA) with the factors MUSCLE (GA, BF, RF, VM, VL, and TA) and LOAD (50%, 62.5%, 75%) for each synergy separately. Post hoc Bonferroni tests were employed to uncover differences in potential main effects and interactions. Sphericity violation was counteracted through Greenhouse–Geisser adjustments. Effect size was evaluated by way of ηp^2^ (Eta partial squared) where 0.01–0.06 constitutes a small effect, 0.06–0.14 a medium effect, and > 0.14 a large effect or Cohen’s d for pairwise post hoc comparisons. All statistical analyses were carried out using JASP version 0.16.1 (University of Amsterdam, Amsterdam, Netherlands) where the significance level was set at *p* < 0.05.

## Results 

**Fig. 2:**
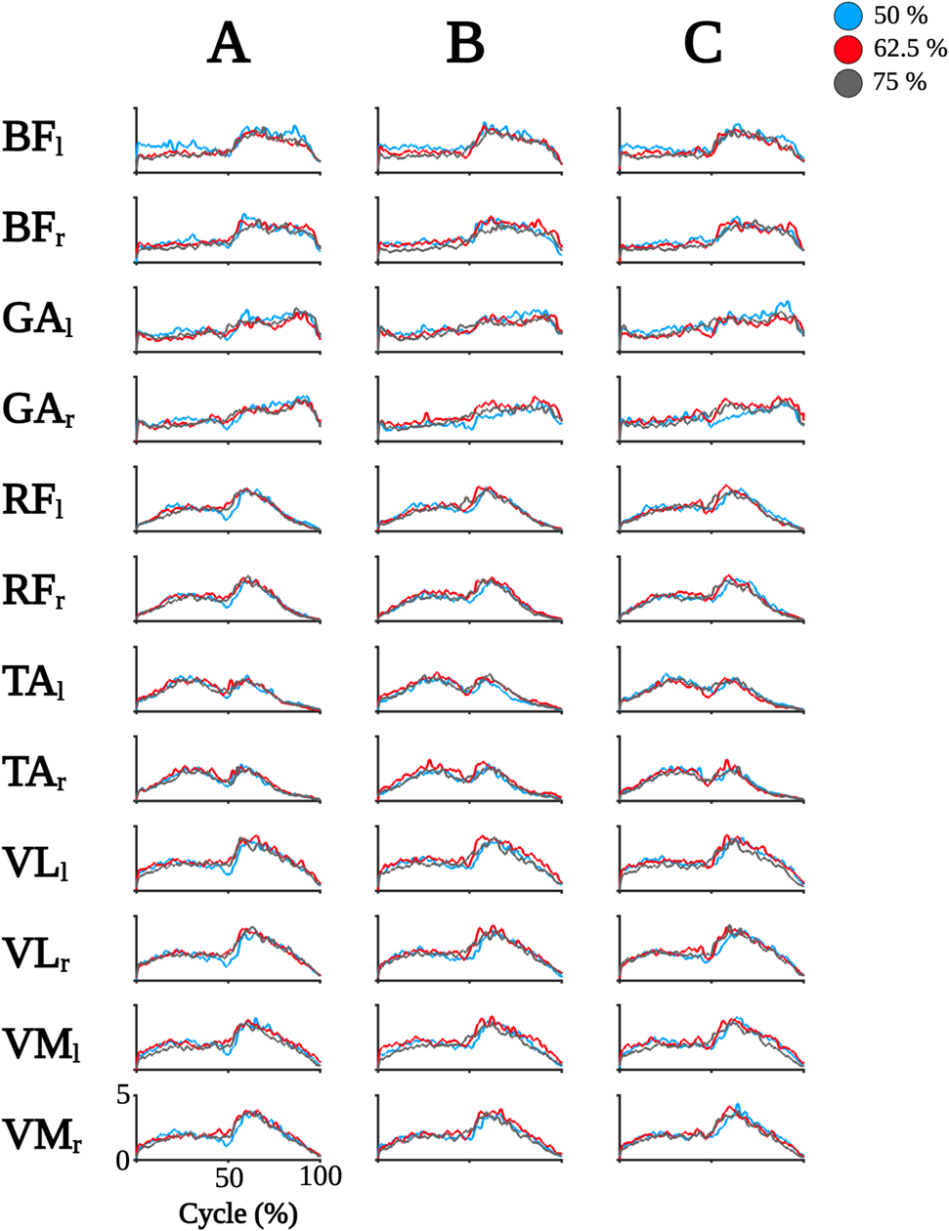
Overview of EMG envelopes. Grand-averaged EMG envelopes are depicted per muscle (rows), set 1 (column A), set 2 (column B), and set 3 (column C) for all loads as indicated in the legend in the upper right corner. Muscles are abbreviated as follows: M. biceps femoris (BF), M. gastrocnemius caput mediale (GA), M. rectus femoris (RF), M. tibialis anterior (TA), M. vastus lateralis (VL), and M. vastus medialis (VM). Left side muscles are abbreviated with a lowercase l, while right side muscles are indicated by lowercase r

First, we inspected EMG envelopes of all muscles to obtain an estimate of the overall variance in activity patterns between sets and loads. EMG envelopes showed consistent patterns across sets and load conditions per muscle (for an overview of EMG envelopes across conditions; please see Fig. 2).

**Fig. 3:**
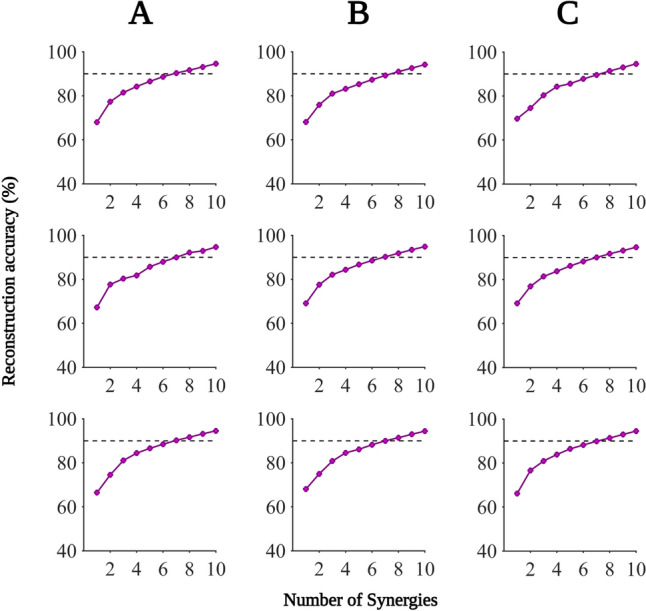
Reconstruction arrays. Reconstruction arrays are highlighted per sets 1–3 (rows 1–3) and loads 50% (**A**), 62.5% (**B**), and 75% of MVC (**C**). Reconstruction array results are shown on average. The defined threshold of 90% is indicated by a black dashed line

Seven synergies accounted for 90.7% ± 1.2% (mean ± standard deviation) of the original EMG envelopes, while an additional synergy contributed less than 3% (Please see Fig. 3 for an overview of reconstruction accuracies across all conditions). For this reason, NNMF was estimated with a fixed number of seven synergies for all participants. The average contribution of synergies 1–7 across all conditions was 12%, 13%, 13%, 13%, 12%, 12%, and 11%, respectively. We subsequently sorted all synergies chronologically, based on the maximum peak observed in their respective waveforms.

**Fig. 4:**
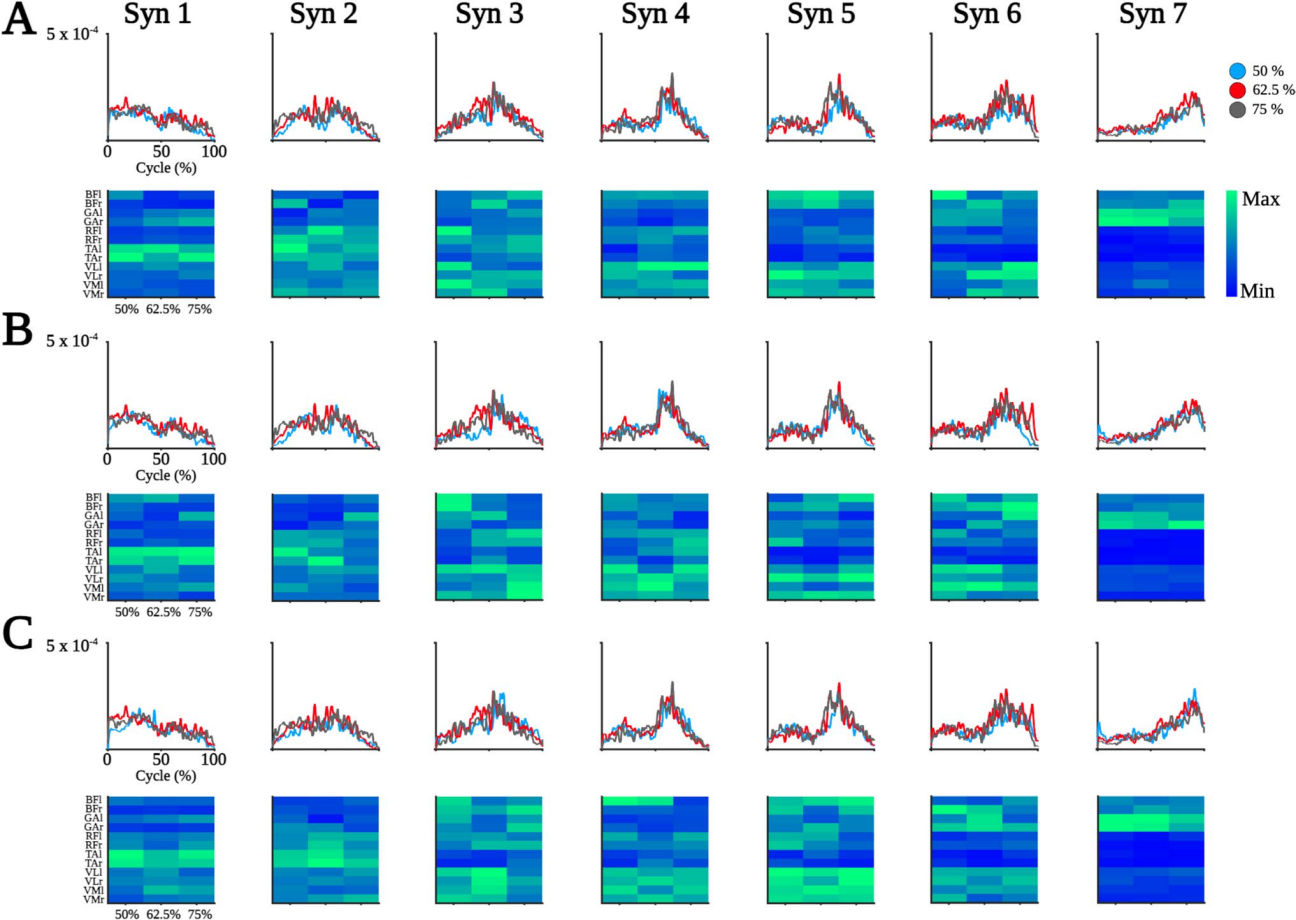
Overview of muscle synergies. Waveforms (upper row) and weights (lower row) are depicted per synergy (columns) and set 1 (**A**), set 2 (**B**), and set 3 (**C**). Colored lines in the waveform plots indicate loads corresponding to the figure legend in the upper right corner. Please note that the magnitudes of weights are scaled per synergy to improve readability. Muscles are abbreviated as follows: M. biceps femoris (BF), M. gastrocnemius caput mediale (GA), M. rectus femoris (RF), M. tibialis anterior (TA), M. vastus lateralis (VL), M. vastus medialis (VM). Left side muscles are abbreviated with a lowercase l while right side muscles are indicated by lowercase r

Considering their temporal structure, the following classification of muscle synergies relates to specific movement periods during the squat, where each SQ_Bp_ cycle consists of three distinctive movement periods: descent, the transition period (TP) between descent and ascent, and ascent. Based on the onset of their peaks, waveforms of Synergy 1 (S1) relate to the descent period. The main peak occurs at the onset of the SQ_Bp_ cycle after which there is a gradual decline, with the exception of a slight increase during TP. Weights of TAr and TAl mainly accounted for the waveform of S1. The main peak of S2 occurs during TP with an increase up to TP and a subsequent decrease in activation. Weights of TAr, TAl, RFr and RFl mainly accounted for the waveform of S2. Waveforms of S3 – S6 all occur during ascent with observable successive peaks after TP, followed by a decrease in activity. Weights of monoarticular (VM and VL) and biarticular (RF and BF) muscles of the thighs mainly accounted for temporal patterns of S3–S6. Generally, the waveform of S7 showed a gradual increase following TP, with the main peak occurring at the end of the ascent period. Weights of GAr and GAl mainly accounted for the waveform of S7. A detailed overview of grand-averaged muscle synergies across sets and loads is provided in Fig. 4.

**Fig. 5:**
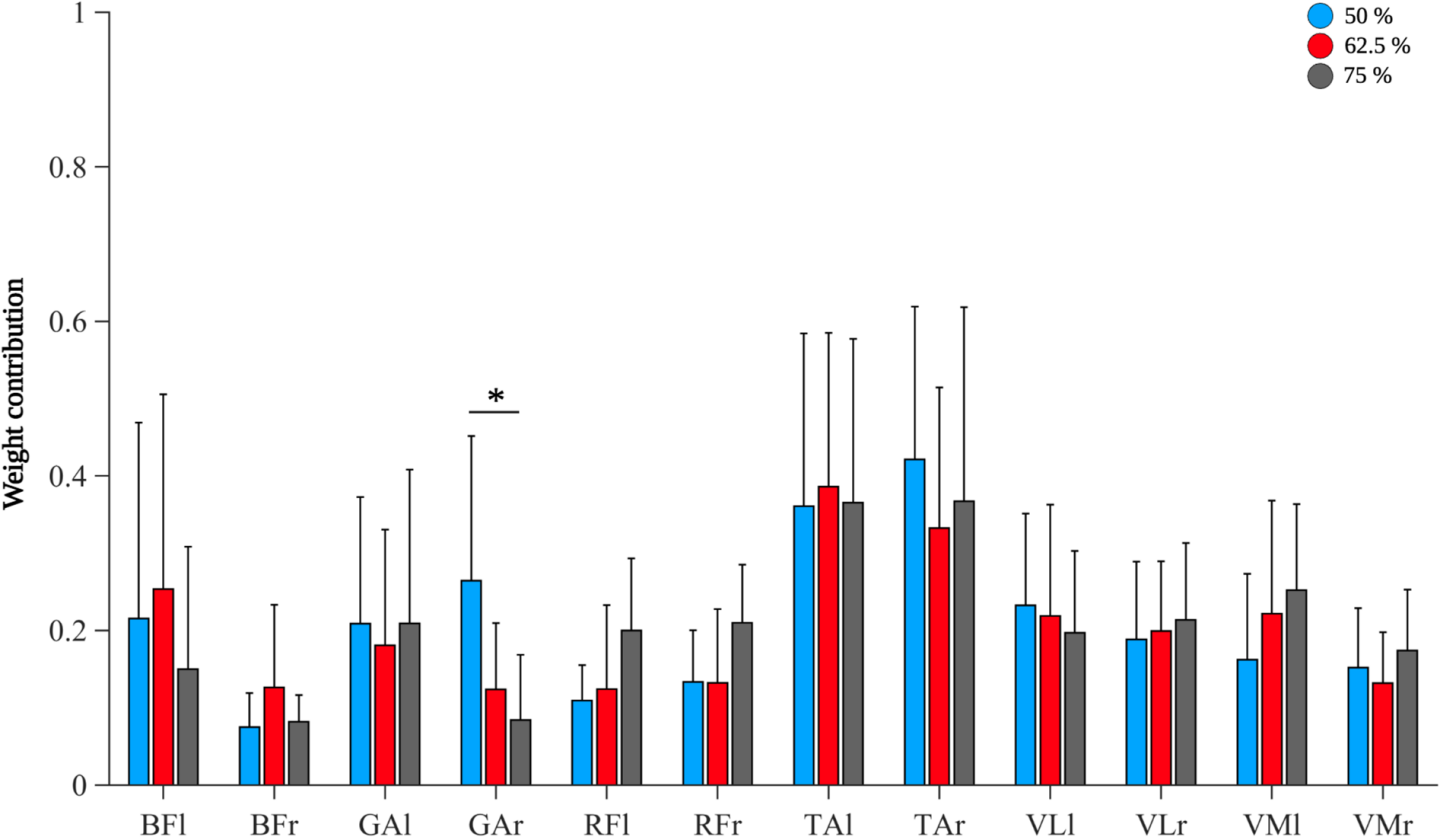
Differences in weight contribution for Synergy 1. Weight contributions of Synergy 1 are depicted per muscle and load. Weights of Synergy 1 were significantly higher for GAr during 50% compared to 75% of 3-RM. Muscles are abbreviated as follows: M. biceps femoris (BF), M. gastrocnemius caput mediale (GA), M. rectus femoris (RF), M. tibialis anterior (TA), M. vastus lateralis (VL), and M. vastus medialis (VM). Left side muscles are abbreviated with a lowercase l, while right side muscles are indicated by lowercase r

All patterns and weights were collapsed across sets to analyze differences in muscle synergies between loads independent of sets. We did not find differences in waveforms of muscle synergies between loads. Comparing weight contributions, we found a significant MUSCLE*LOAD interaction (F_(22,198)_ = 1.897, *p* = 0.012, ηp^2^ = 0.174) for S1, where post hoc comparisons revealed a significant difference in weight contribution of GAr between 50 and 75% (MD = 0.180, SE = 0.044, *p* = 0.043, *d* = 1.182) (Fig. 5).

## Discussion 

With the present study, we aimed to evaluate coordinative demands during SQ_Bp_ performance as a function of external load. For this purpose, we analyzed differences in muscle synergy properties between various load levels. Our findings revealed seven muscle synergies to account for 90% of the variance in multi-muscle EMG data during SQ_Bp_ for all load levels. Waveforms of muscle synergies did not differ between load levels. Analysis of synergy contribution showed a significant difference in the synergy contribution of GAr between the 50% and 75% load levels for S1. However, no other differences relating to synergy contribution were observed. All findings and their implications are discussed below.

On a functional level, the synergies obtained in this study correspond to biomechanical profiles previously associated with SQ_Bp_. A single SQ_Bp_ cycle comprises periods of descent, TP, and ascent. During descent, TA, VL, and RF are primarily involved (Robertson et al. 2008). Descent is initiated by TA activity, presumably to provide joint stability in the ankles during early dorsiflexion (Alves et al. 2009). Here, we observed TA to primarily account for S1, supporting the assumption that TA activity initiates SQ_Bp_. Dorsiflexion during descent is accompanied by subsequent knee flexion. Among the muscles recorded in this study, BF and GA are responsible for knee flexion (Escamilla 2001), which explains the additional input of BF and GA to S1. The observed difference in weight contribution of GAr to S1 between 50 and 75% load levels may be explained by the brevity of GA activity during descent. In short, GA unlocks the knee to enable knee flexion through a brief burst in activity (Robertson et al. 2008), the duration of which can vary between loads, potentially leading to a significant difference in weight contribution for GAr to S1. The descent period connects to TP, which functions as a transition period between descent and ascent to enable sufficient counter-movements to support the ascent period. The prime movers during TP are VL, VM, and RF (Robertson et al. 2008). During TP, knee extensors, specifically VM and VL, control knee flexion and ultimately terminate the descent period. Both muscles are gradually supported by RF in the last part of descent until maximum SQ_Bp_ depth (Robertson et al. 2008). Here, strong contributions from TA and RF to S2 indicate that the ascent phase has not yet commenced, whereas marginal input from VM and VL to S2 may reflect the eccentric work of these muscles during the latter part of the descent and TP. The subsequent ascent period was constructed by four synergies (S3–S6). Generally, ascent during SQ_Bp_ involves the activation of numerous hip extensors, knee extensors and ankle flexors (Robertson et al. 2008). Concurring with this, S3–S6 contributions mainly come from monoarticular (VM and VL) and biarticular (RF and BF) upper thigh muscles as well as GA. While VM and VL provide most of the concentric work, RF, BF, and GA contract eccentrically during the initial part of ascent (Escamilla 2001). When the knee is extended during ascent, the force acting on BF is directed to the hip, thereby supporting hip extension. Since the lever arm of the BF is smaller at the knee than at the hip, the flexion moment at the knee is smaller than the extension moment at the hip for the same force in the muscle leading to a hip extension during ascent of SQ_Bp_ (Bryanton et al. 2012). This interplay between VM, VL, RF, and BF is at the core of S3–S6 weight contribution, while being supported by GA which progressively increases relative input until S7, the end of the ascent period, where GA is the main contributor. Previous reports show GA to produce the largest power at the end of ascent during SQ_Bp_, supporting our findings (Robertson et al. 2008). The observed order of recruitment and relative contribution from hip extensors to knee extensors, and finally ankle flexors during ascent can also be observed in related movements such as counter-movement jumps (Nagano et al. 1998) or sit-to-stand tasks (Janssen et al. 2002), and might imply naturally stabilized movement patterns following lower extremity compound movement acquisition.

We did not observe any significant differences in muscle synergy waveforms between load conditions. This finding is consistent with contemporary evidence on the influence of a range of external constraints on muscle synergies. For example, previous research in animals demonstrated that muscle synergies underlying various natural movements remain constant across loading conditions (Cheung et al. 2009). These findings were subsequently extended to human populations. Using a variety of tasks, including variable arm weight support during reaching movements (Coscia et al. 2014), and force adaptation tasks in the arm muscles under both isometric (Roh et al. 2012) and dynamic conditions (Roh et al. 2019), several studies observed stable muscle synergy properties across loading conditions during simple movements. Similar results were found when studying more compound movements such as cycling at different mechanical constraints (Hug et al. 2011), and rowing at different intensities (Turpin et al. 2011). Taken together, these results imply that load does not significantly affect motor control strategies related to spatiotemporal composition of both simple and compound movements. It can be assumed that the application of external load changes joint torque dynamics across the body (Cheung et al. 2009). During SQ_Bp_, hip extensor, knee extensor and plantar flexor torques as well as corresponding EMG activity increase as a function of load, with the greatest increases observed in hip extensors (Bryanton et al. 2012). It follows that muscle synergies may reflect spatiotemporal instructions to goal-directed movements that are subject to little, if any, influence from dynamic control parameters, e.g., torques altered by external loads or differences in sensory information flow (Cheung et al. 2009). This appears to be a sensible assumption, particularly in view of the consistency of synergy waveforms between load conditions.

### Limitations

In principle, muscle synergy analyses are affected by the number of muscles studied as well as the determination of the appropriate number of synergies. Here we studied 12 muscles, motivated by the fact that we focused on muscle synergies between lower extremity muscles during SQ_Bp_. Future studies should explore the contributions of upper body (e.g., trapezius) and trunk muscles (e.g., erector spinae) to evaluate synergistic relationships between upper and lower body musculature during compound movements. Furthermore, in this study, we examined SQ_Bp_ in the smith machine. In comparison, a free weight squat features more degrees of freedom, which raises the question of how muscle synergies differ between these two related compound movements. Previous studies observed EMG activity to be significantly elevated during free weight squats for several prime movers, further suggesting muscular interplay to differ between both movements (Schwanbeck et al. 2009). Future studies should, therefore, study the synergistic composition of both movements to uncover potential differences pertaining to the number of degrees of freedom between these two related compound movements. Another limitation relates to the degree of dimensionality reduction, i.e., the amount of muscle synergies underlying multi-channel EMG data. This variability between studies may be due to several factors, such as the muscles analyzed, differences in preprocessing, and the applied factorization method (Coscia et al. 2014). We have addressed these issues by aligning our processing pipeline with recommendations from current literature (Turpin et al. 2021). An additional factor contributing to the reduction in dimensionality is the presence of muscle-specific synergies. In this study, S1 and S7 may represent synergy vectors dominated by a single muscle group. The interpretation of these synergies is still a matter of debate. Some authors argue that muscle-specific synergies reflect specifically tailored central nervous motor commands, whereas others attribute them to differences in the factorization algorithm used (Coscia et al. 2014). In either case, research is needed to further explore the mechanisms underlying muscle-specific synergies.

## Conclusion

In conclusion, we provide the first account of muscle synergies during SQ_Bp_ performance at different load levels. Muscular coordination during SQ_Bp_ appears not to change significantly as a function of load, since the basic movement composition is preserved across various external loads. Learning proper SQ_Bp_ technique is therefore crucial to ensure functionally adequate SQ_Bp_ performance, while enabling different external loads to be efficiently addressed. Furthermore, proficient execution of fundamental movements such as the SQ_Bp_ holds direct implications for a variety of sports as well as rehabilitative settings. Early learning of such movements can, therefore, be beneficial both prospectively, in terms of building a functional motor repertoire, and preventively, in terms of injury protection.

## Data Availability

The datasets generated during and/or analyzed during the current study are available in the Figshare repository (https://figshare.com/articles/dataset/Data_-_SquatSyn/21995144).
